# Factors influencing use of community treatment orders and quality of care that people receive: results of a national survey in England and Wales

**DOI:** 10.1192/bjb.2019.23

**Published:** 2019-10

**Authors:** Harry Lei, Kirsten Barnicot, Emily Maynard, Angela Etherington, Krysia Zalewska, Alan Quirk, Rahil Sanatinia, Stephen J. Cooper, Mike J. Crawford

**Affiliations:** 1 Imperial College London; 2 Royal College of Psychiatrists, London

**Keywords:** Community treatment order, psychosis, community care, mental health act

## Abstract

**Aims and method:**

We conducted a secondary analysis of data from the National Audit of Psychosis to identify factors associated with use of community treatment orders (CTOs) and assess the quality of care that people on CTOs receive.

**Results:**

Between 1.1 and 20.2% of patients in each trust were being treated on a CTO. Male gender, younger age, greater use of in-patient services, coexisting substance misuse and problems with cognition predicted use of CTOs. Patients on CTOs were more likely to be screened for physical health, have a current care plan, be given contact details for crisis support, and be offered cognitive–behavioural therapy.

**Clinical implications:**

CTOs appear to be used as a framework for delivering higher-quality care to people with more complex needs. High levels of variation in the use of CTOs indicate a need for better evidence about the effects of this approach to patient care.

Community treatment orders (CTOs) were introduced in England and Wales in 2008, in an attempt to reduce the use of in-patient services for patients with poor adherence to their treatment.^1–3^ They require individuals with mental disorders who have been detained in hospital to adhere to treatment and supervision in the community. Non-compliance with CTOs may lead to revocation, where the patient is involuntarily admitted back into hospital for further treatment. The use of similar legislation to enforce community treatment also exists in other countries, including the USA, Australia and New Zealand.^4,5^

Although CTOs have been recommended as a method for improving adherence and patient safety, patients treated under CTOs feel more coerced.^6^ The use of CTOs has been much higher than was initially anticipated,^7,8^ and has increased considerably since they were first introduced.^8,9^ Concerns have been raised about greater use of compulsion amongst people from ethnic minority communities.^10–12^ Data submitted to NHS Digital in 2016 indicated that Black or Black British patients were almost nine times more likely to be treated on a CTO than White British patients.^13^ A systematic review of data from 38 studies of clinical practice in the UK, which compared the use of in-patient mental health services by different ethnic groups, found that Black patients were over four times more likely to be admitted to hospital on a compulsory basis than White patients.^10^ However, other studies have reported that associations between use of compulsory treatment and ethnicity may be reduced or eliminated when other sociodemographic and clinical factors are taken into account.^14,15^ To date, the influence of clinical and sociodemographic factors on the association between ethnicity and use of CTOs has not been examined.

There is considerable variation in the experiences of people treated under CTOs.^6^ Surveys of both patients and carers indicate that many believe their main aim is to try to force people to take regular mediation.^6,16^ It has been argued that use of CTOs can improve a person's mental health and quality of life, but negative findings of randomised trials^17,18^ and limited information about the quality of care that people actually receive have led to calls for further research in this area.^11,19^

The National Clinical Audit of Psychosis is a 10 year programme of work commissioned by the Healthcare Quality Improvement Partnership as part of the National Clinical Audit and Patient Outcomes Programme in England and Wales, which aims to improve the quality of care that people with psychosis receive.^20^ In the light of concerns about increasing use of CTOs, patient representatives from the Steering Committee that oversees this programme of work requested that data on use of CTOs be included in the third round of the audit. By collecting data on whether people in the audit were being treated using a CTO, we aimed to identify patient characteristics that predict use of CTOs in England and Wales and to explore the quality of care received by people who were treated under a CTO compared with those who were not.

## Method

This study was based on a secondary analysis of data from the third round of the National Clinical Audit of Psychosis.^21^ Data for the audit were obtained from clinical records of patients between September and November 2017. The study population were patients aged 16 or over who had received care from a provider of National Health Service (NHS) mental health services for at least 12 months on a census date (1 July 2017). To take part in the audit a patient had to have a current ICD-10 diagnosis of psychotic disorder secondary to alcohol or substance abuse, schizophrenia, persistent delusional disorder, schizoaffective disorder or other non-organic or unspecified psychotic disorders made before the age of 60 and 12 months or longer before the census date.^22^

All providers of mental health services in England and Wales were helped to generate a random sample of eligible patients. The sample size for each trust was between 100 and 300 patients, depending on the size of the trust.^22^ For each selected patient, staff working in the trust extracted data from electronic and other patient records to complete a 49-item online data collection form. This form included questions on demographic factors (including age, gender and ethnicity), clinical factors (including whether the patient had received in-patient treatment during the previous 12 months and whether the patient was currently in remission), data on the physical and mental healthcare that the patient had received, and scores from the Health of the Nation Outcome Scales (HoNOS) completed during the previous 12 months.^22^ HoNOS is a clinician-rated outcome measure which assesses patients' difficulties in a variety of mental health domains during the 2 weeks leading up to the point of rating. Scores for each domain range from 0 (absent) to 4 (severe), with higher scores indicating greater impairment.^23^ Finally, we collected data from a single item that asked whether the patient was currently being treated on a CTO.

Prior to the start of the audit, the National Research Ethics Service and the Ethics and Confidentiality Committee of the National Information Governance Board advised that formal ethical approval was not required for this quality improvement initiative. Approval for the secondary analysis of the data in this study was obtained from the Healthcare Quality Improvement Partnership prior to the start of data analysis.

### Data management and analysis

We excluded data from people who were in-patients at the point when data were collected, because it was unclear whether or not such patients had been treated using a CTO prior to their admission. For this analysis, we extracted data from the audit on demographic, clinical and service factors, together with information about whether the patient was currently being treated using a CTO, and evaluated the association between CTO treatment and demographic, clinical and service provision factors. Among all patients being treated in the community, we calculated the proportion of people currently being treated on a CTO, together with 95% confidence intervals.

We used χ^2^ tests and Mann–Whitney *U*-tests to compare the demographic and clinical characteristics of patients treated under CTOs with those of patients who were not. We then used univariate ordinal logistic regression to compare HoNOS ratings between patients who were treated under CTOs and those who were not. Demographic and clinical variables identified as differing significantly between patients treated under CTOs and others (*P* < 0.05) were then entered into a multivariate logistic regression in order to determine which variables were independently associated with a greater likelihood of treatment under a CTO. The multivariate logistic regression used multilevel modelling with a random effect for NHS trust in order to adjust for differences between NHS trusts in frequency of CTO usage.

Differences in service provision between patients who were treated under a CTO and those who were not were examined using multilevel logistic regression, adjusting for potential demographic and clinical confounders (age, sex, ethnicity, in-patient psychiatric hospital admission in the past 12 months), and including a random effect for patients' NHS trust in order to adjust for differences between NHS trusts in service provision. We compared 15 aspects of the quality of physical and mental healthcare that people received: whether the patient was prescribed a single antipsychotic drug; whether the current daily dose of antipsychotic medication was within the upper limits recommended in the British National Formulary;^24^ whether the patient had ever been offered cognitive–behavioural therapy for psychosis (CBTp); whether patients in contact with their families had been offered family intervention; whether the patient had been screened for smoking, harmful alcohol use, and substance misuse; whether interventions were offered for smoking cessation, or alcohol or substance use to those requiring them; whether the patient had a current care plan; whether there was documented evidence that the patient had been given information about how to contact healthcare services if in crisis; whether the patient had been offered support to obtain employment; and whether or not there was documented evidence that any identified carer had been offered an assessment of their needs.

All statistical analysis was carried out using SPSS version 21 or Stata SE version 14.2.^25,26^

## Results

All 62 trusts and health boards in England and Wales took part in the audit. Data were collected from 9449 patients, of whom 8760 patients were being treated in the community and were therefore eligible for this study. Table 1 lists the frequency of use of CTOs at a national and regional level. Across England and Wales, 6.04% (*n* = 529) of patients were being treated on a CTO at the time of the audit. The proportion of people being treated on a CTO was highest in Wales and lowest in the South West of England. There was also considerable variation in use of CTOs among patients treated across the 62 trusts and health boards, ranging from 1.06 to 20.22%.

**Table 1:** Number and proportion of people with psychosis on a CTO in England and Wales

Area	*N*	On CTO	Not on CTO	% on a CTO	95% CI
Total	8760	529	8231	6.04	(5.55–6.53)
London	1514	92	1422	6.08	(4.88–7.28)
North East	2140	135	2005	6.31	(5.28–7.33)
North West	1244	95	1149	7.64	(6.16–9.11)
South East	1973	113	1860	5.73	(4.71–6.75)
South West	1492	63	1429	4.22	(3.20–5.24)
Wales	397	31	366	7.81	(5.17–10.45)

### Predictors of use of CTOs

Demographic and clinical characteristics of patients who were and were not treated under a CTO are presented in Table 2, and HoNOS scores of the two groups are presented in Table 3. Among the 6853 White British patients, 399 (5.82%) were currently being treated on a CTO; among the 788 patients whose ethnicity was recorded as Black or Black British, 71 (9.02%) were currently being treated on a CTO. Scores on all but four HoNOS items were higher among those treated on a CTO compared to those who were not. Differences in the use of CTOs across trusts explained 5.0% of the overall variance in use of CTOs (95% C.I. 2.70–8.77). In the multivariate multilevel model using a random effect for NHS trust, the variables significantly independently predicting a greater likelihood of CTO use were: younger age, male gender, psychiatric hospital admission in the past 12 months, current substance misuse, and greater levels of cognitive problems on the HoNOS (Table 4).

**Table 2:** Comparison of demographic and clinical characteristics of patients treated and not treated on a CTO

Variable	Treated on CTO		Not treated on CTO		*P*-value (Z or χ^2^)
	Mean/*n*	%	Mean/*n*	%	
Mean age in years (s.d.)	44.05	11.51	46.84	11.35	<0.001* (5.450)
Gender					0.013* (8.733)
Male	367	69.38	5334	64.80	
Female	161	30.43	2895	35.17	
Other/undefined	1	0.19	2	0.02	
Ethnicity					0.001* (19.910)
White	399	75.43	6454	78.41	
Black or Black British	71	13.42	717	8.71	
Asian or British Asian	26	4.91	640	7.78	
Mixed	17	3.21	192	2.33	
Other ethnic groups	16	3.02	228	2.77	
Diagnosis					<0.001* (19.914)
F10–19 Substance-induced	17	3.21	230	2.79	
F20 Schizophrenia	368	69.57	5946	72.24	
F25 Schizoaffective disorder	118	22.31	1341	16.29	
F22, F24, F28, F29 Other	26	4.91	714	8.68	
Time since diagnosis					0.654 (0.201)
Less than 3 years	103	19.47	1538	18.69	
3 years or longer	426	80.53	6693	81.31	
Current mental health					<0.001* (63.464)
Full remission	81	15.31	2088	25.37	
Partial remission – minimal symptoms	255	48.20	4258	51.73	
Partial remission – substantial symptoms	165	31.19	1529	18.58	
Not in remission	28	5.29	356	4.33	
Alcohol consumption					0.852 (0.035)
Harmful use	67	29.26	875	28.68	
No harmful use	162	70.74	2176	71.32	
Substance misuse					<0.001* (96.656)
Yes	159	33.40	1229	17.46	
No	317	66.60	5810	82.54	
Use of in-patient services					<0.001* (391.357)
Admitted in past 12 months	224	42.3	975	11.9	
No admission	305	57.7	7526	88.2	

* *P* < 0.05.

**Table 3:** HONOS scores of 5960 patients according to whether treated on a CTO

HONOS item	Score	On a CTO *N* = 347 *n* (%)	Not on a CTO *N* = 5613 *n* (%)	Odds ratio	95% CI	*P*-value
Overactive, aggressive, disruptive or agitated behaviour	0	130 (37)	3062 (55)	1.98	1.63–2.41	<0.001
	1	99 (29)	1382 (25)			
	2	80 (23)	810 (14)			
	3	25 (7)	301 (5)			
	4	13 (4)	58 (1)			
Non-accidental self-injury	0	298 (86)	4820 (86)	1.03	0.76–1.41	0.834
	1	23 (7)	474 (8)			
	2	15 (4)	201 (4)			
	3	8 (2)	91 (1.5)			
	4	2 (1)	24 (0.5)			
Problem drinking or drug-taking	0	192 (55)	3897 (69)	1.85	1.50–2.28	<0.001
	1	44 (13)	627 (11)			
	2	54 (16)	523 (9)			
	3	40 (11)	412 (7)			
	4	16 (5)	135 (2)			
Cognitive problems	0	148 (43)	2934 (52)	1.52	1.25–1.86	<0.001
	1	91 (26)	1432(26)			
	2	76 (22)	904 (16)			
	3	29 (8)	287 (5)			
	4	3 (1)	49 (1)			
Physical illness or disability problems	0	164 (47)	2448 (44)	0.88	0.72–1.07	0.192
	1	72 (21)	1227 (22)			
	2	70 (20)	1168 (21)			
	3	35 (10)	620 (11)			
	4	6 (2)	134 (2)			
Problems associated with hallucinations and delusions	0	54 (16)	1453 (26)	1.92	1.60–2.34	<0.001
	1	68 (20)	1156 (21)			
	2	97 (28)	1782 (32)			
	3	90 (26)	975 (17)			
	4	36 (10)	231 (4)			
Problems with depressed mood	0	133 (39)	2235 (40)	1.01	0.83–1.23	0.923
	1	112 (32)	1731 (31)			
	2	79 (23)	1251 (22)			
	3	17 (5)	355 (6)			
	4	3 (1)	34 (1)			
Other mental and behavioural problems	0	97 (28)	1700 (30)	1.07	0.88–1.30	0.496
	1	71 (21)	1063 (19)			
	2	98 (29)	1655 (30)			
	3	60 (17)	1005 (18)			
	4	16 (5)	167 (3)			
Problems with relationships	0	67 (19)	1835 (33)	2.08	1.71–2.53	<0.001
	1	78 (23)	1452 (26)			
	2	101 (29)	1458 (26)			
	3	83 (24)	739 (13)			
	4	16 (5)	113 (2)			
Problems with activities of daily living	0	86 (25)	1845 (33)	1.43	1.18–1.73	<0.001
	1	80 (23)	1366 (24)			
	2	108 (31)	1, 513(27)			
	3	60 (18)	762 (14)			
	4	10 (3)	120 (2)			
Problems with living conditions	0	188 (55)	3633 (65)	1.58	1.28–1.95	<0.001
	1	72 (21)	973 (17)			
	2	44 (13)	622 (11)			
	3	29 (8)	269 (5)			
	4	12 (3)	91 (2)			
Problems with occupation and activities	0	99 (29)	2063 (37)	1.47	1.21–1.79	<0.001
	1	74 (22)	1246 (22)			
	2	97 (28)	1426 (25)			
	3	60 (17)	706 (13)			
	4	15 (4)	153 (3)

**Table 4:** Multivariate logistic regression for sociodemographic and clinical predictors of CTO use

Independent variable^a^	Odds ratio	95% CI	*P*-value
Age	0.98	0.96–1.00	0.049
Gender: Male	1	–	–
Female	0.48	0.28–0.80	0.005
Ethnicity: White	1	–	–
Black or Black British	1.35	0.77–2.36	0.297
Asian or British Asian	0.76	0.28–2.04	0.587
Mixed	0.99	0.36–2.75	0.983
Other ethnic groups	1.82	0.52–6.35	0.347
Diagnosis: Other psychoses	1	–	–
Schizophrenia	1.06	0.68–1.65	0.790
Mental health: Partial/ no remission	1	–	
Full remission	0.68	0.38–1.22	0.203
Harmful alcohol consumption: No	1	–	–
Yes	0.74	0.47–1.16	0.186
Substance misuse: No	1	–	–
Yes	1.98	1.28–3.05	0.002
Psychiatric hospital admission: No	1	–	–
Yes	4.57	3.07–6.79	<0.001
HONOS Overactive, aggressive, behaviour	1.05	0.85–1.28	0.661
HONOS Problem drinking or drug-taking	0.93	0.78–1.10	0.388
HONOS Cognitive problems	1.28	1.04–1.57	0.021
HONOS Problems with hallucinations and delusions	1.08	0.90–1.30	0.397
HONOS Problems with relationships	1.21	1.02–2.08	0.080
HONOS Problems with activities of daily living	0.94	0.75–1.18	0.597
HONOS Problems with living conditions	1.07	0.88–1.30	0.484
HONOS Problems with occupation and activities	0.93	0.77–1.14	0.509

a. Adjusted for NHS trust.

### Quality of care delivered to patients on CTOs

Data on the quality of care provided to patients who were and were not being treated on a CTO are presented in Table 5. After adjusting for age, sex, ethnicity and history of in-patient psychiatric hospital admission in the past 12 months, patients treated on a CTO were more likely to be screened for smoking and substance misuse, have a current care plan, have documented evidence of being given contact details to be used in a crisis, and be offered CBTp.

**Table 5:** Descriptive statistics and logistic regression comparing quality of care received by patients on a CTO and those who were not

Aspect of care^a^	On CTO *N* = 529		Not on CTO *N* = 8231		Odds ratio	95% CI	*P*-value
	*n*	%	*n*	%			
Was screening for smoking carried out?					1.61	1.14–2.30	0.008
Yes	490	92.63	7114	86.43			
No	37	7.37	1117	13.57			
Was screening for alcohol misuse carried out?					1.35	0.95–1.93	0.096
Yes	490	92.63	7261	88.22			
No	39	7.37	970	11.78			
Was screening for substance misuse carried out?					1.49	1.03–2.15	0.032
Yes	493	93.19	7203	87.51			
No	36	6.81	1028	12.49			
Was intervention for smoking offered if needed?^b^					1.30	0.94–1.80	0.108
Offered	272	81.19	3060	78.7			
Not offered	63	18.81	830	21.34			
Was intervention for alcohol offered if needed?^b^					1.00	0.41–2.44	0.999
Offered^c^	60	89.55	775	88.57			
Not offered	7	10.45	100	11.43			
Was intervention for substance misuse offered?^b^					1.05	0.64–1.74	0.834
Offered^c^	133	83.65	1022	83.16			
Not offered	26	16.35	207	16.84			
Polypharmacy being used					0.84	0.65–1.08	0.163
Yes	82	15.59	1440	18.22			
No	444	84.41	6464	81.78			
Total antipsychotic drug dose above BNF maxima					0.79	0.54–1.16	0.228
Yes	32	6.08	573	7.25			
No	494	93.92	7331	92.75			
Evidence of patient involvement in deciding antipsychotic medication					1.05	0.86–1.28	0.648
Yes	351	66.73	5181	65.55			
No	175	33.27	2723	34.45			
Current care plan					2.97	1.71–5.18	<0.001
Yes	514	97.16	7597	92.30			
No	15	2.84	634	7.70			
Crisis contact details provided					1.62	1.15–2.26	0.005
Yes	484	91.49	7183	87.27			
No	45	8.51	1048	12.73			
Offered CBTp?					1.27	1.03–1.56	0.026
Yes	168	31.76	2144	26.05			
No	361	68.24	6087	73.95			
Offered family intervention^d^					1.24	0.86–1.78	0.248
Yes	66	39.76	796	32.77			
No	100	60.24	1633	67.23			
Offered work support programme^e^					0.53	0.16–1.83	0.317
Yes	12	63.16	242	76.34			
No	7	36.84	75	23.66			
Evidence that carer's needs were assessed^f^					1.30	0.98–1.74	0.073
Yes	145	61.97	1987	54.02			
No	89	38.03	1691	45.98			

a. All associations adjusted for age, gender, ethnicity, hospital admission in the past 12 months and NHS trust.

b. Based on subsample of 4225 individuals recorded as current smokers, 942 were identified as current hazardous drinkers and 1388 were identified as having a current substance misuse problem.

c. Of the 3332 smokers offered a smoking intervention, 1508 refused this (45.26%); of the 835 hazardous drinkers offered an alcohol intervention, 186 refused this (19.75%); of the 1155 substance abusing individuals offered an intervention, 288 refused this (20.75%).

d. Based on subsample of *n*  =  2595 cases where the patient was in contact with their family and allowed the clinical team to contact them, and where a family therapist was available and family intervention had not been deemed inappropriate for the patient.

e. Based on subsample of 336 cases where it was documented that the patient was seeking work.

f. Based on subsample of 3912 cases where it was documented that the patient had a carer.

## Discussion

The results of this study suggest that, in England and Wales, age, gender, coexisting substance misuse and problems with cognition predict whether people are treated on a CTO. Previous studies have reported higher rates of substance misuse among people who are treated in the community on a compulsory basis.^27^ People who have substance misuse problems in addition to psychosis are more likely to relapse and more likely to be admitted to hospital.^28,29^ Data from this study suggest that clinicians use CTOs to provide a framework for trying to mitigate against these risks.

By contrast, previous studies of compulsory community treatment have not examined the influence of cognitive impairment on the use of CTOs. Problems with cognition are defined in the HoNOS as ‘problems of memory, orientation and understanding associated with any disorder: learning disability, dementia, schizophrenia’.^23^ We did not collect data on whether people in the audit had other coexisting mental health conditions. However, whether the higher levels of cognitive problems rated on the HoNOS among those treated on CTOs resulted from coexisting intellectual impairment or were directly associated with their psychosis,^30^ it seems that clinicians are more likely to use CTOs when patients with psychosis also have difficulties with making decisions and organising their lives. Surveys of clinicians in both the UK and New Zealand indicate that the most important role of CTOs is ensuring that patients maintain their contact with mental health services.^31,32^ Clinical teams may judge that the power of recall helps ensure that people with psychosis who have impaired cognition maintain their contact with services.

We did not find evidence that ethnicity was an independent predictor of the use of CTOs. While Black and Black British patients were 55% more likely to be treated on a CTO than White British patients, differences in the adjusted odds of being treated on a CTOs among ethnic groups were not statistically significant. Nor did we find evidence that other aspects of mental state, including suicidal behaviour or problems associated with hallucinations and delusions, predicted use of CTOs.

### Study strengths and limitations

The main strength of this study is that we were able to analyse data on patients from all trusts in England and Wales, which means that we can be confident that these findings are generalisable across the country. Another strength is that there was little missing data. The items with the largest amount of missing data were scores on the HoNOS, but even these scores were available for 70% of study patients. The inclusion of HoNOS data was important as it enabled us to examine whether demographic factors truly influence the use of CTOs once the potentially confounding effects of clinical variables have been taken into account.

The main limitation of the study is the reliance on data which were retrospectively extracted from patient records. This means that if interventions were delivered but not documented, these would not have been included in our analysis. Various steps were taken to maximise the reliability of data gathered in the audit, including piloting the data collection tool during an earlier round of the audit and providing comprehensive guideline notes for those tasked with extracting and entering data on the audit's online data management platform. Although questions have been raised about the reliability of HoNOS,^33^ it remains the only outcome measure in widespread use in the UK and therefore provides a valuable source of routine data on the health and social functioning of people who use secondary care mental health services.^34,35^ We were able to analyse data from a large sample of over 8000 patients. However, the relatively small numbers of patients on a CTO meant that we had limited power to examine differences in use of CTOs among some groups of patients. Although we are only able to report on the use of CTOs among people with schizophrenia and related psychoses, most people treated using a CTO have psychosis. It is important that future research examine factors that influence the use of CTOs among all those who may be treated under them, including people with intellectual disability.

### Implications of study findings

The results of this study provide some assurance about the quality of care that people treated using CTOs in England and Wales receive. Rather than simply being a means to encourage adherence to medication or attend follow-up appointments, people on CTOs in this study were more likely to be referred for psychological therapy and to have documented evidence of care plans and assessment of their physical health. Although many patients treated under CTOs believe that they are used to enforce use of medication,^6^ the results of this study indicate that people on CTOs may be receiving better quality of care overall. Nonetheless, it is important to note that even among people on CTOs, standards of care, especially in relation to psychological treatments and physical health assessment, fall below recommended standards.^36^

Arguably the most important finding in this study is the high degree of variation in use of CTOs across different trusts and in different parts of the country. CTOs represent a significant limitation on the rights of patients, and the high level of variation is therefore of concern.^12^ There are a number of possible explanations for this variation. These include random variation resulting from study sampling, differences in the populations served by different trusts and differences in the organisation and delivery of local services. Trevithick and colleagues noted that trusts serving large urban centres have higher rates of CTO use.^8^ Studies have also shown that there are higher rates of CTO use in trusts that provide specialist services for people with intellectual disability.^8,31^

Another reason for differences in levels of use of CTOs in different parts of the country could be continuing uncertainty about the clinical effectiveness of this approach to helping people with psychosis. While the results of the OCTET trial and other randomised trials of compulsory community treatment suggest that CTOs do not reduce use of in-patient services or lead to improved mental health or quality of life,^17,18^ questions have been raised about whether the results of these trials can be generalised to routine clinical settings^37^ and many psychiatrists remain convinced of their benefit. It seems likely, given this degree of uncertainty, that the extent to which different teams use CTOs is influenced by the opinions and experiences of clinicians who are responsible for delivering patient care.

## Conclusions

The findings of this study suggest that, in addition to marked variation at the trust level, male gender, younger age, coexisting substance misuse and problems with cognition influence the use of CTOs in England and Wales. Patients treated on CTOs appear to be receiving higher quality of care than those who are treated on a voluntary basis. These results highlight the need for further research into the costs and benefits of treating patients using CTOs so that unwarranted variation in their use can be minimised.

## About the authors

**Harry Lei** is an Undergraduate Medical Student in the Centre for Psychiatry, Imperial College, London, UK. **Kirsten Barnicot** is a Research Fellow in the Centre for Psychiatry, Imperial College, London, UK. **Emily Maynard** is a Project Officer at the College Centre for Quality Improvement, Royal College of Psychiatrists, London, UK. **Angela Etherington** is a Service User Advisor at the College Centre for Quality Improvement, Royal College of Psychiatrists, London, UK. **Krysia Zalewska** is a Project Manager at the College Centre for Quality Improvement, Royal College of Psychiatrists, London, UK. **Alan Quirk** is a Senior Project Manager at the College Centre for Quality Improvement, Royal College of Psychiatrists, London, UK. **Rahil Sanatinia** is a Research Fellow in the Centre for Psychiatry, Imperial College, London, UK. **Stephen J. Cooper** is a Clinical Advisor at the College Centre for Quality Improvement, Royal College of Psychiatrists, London, UK. **Mike J. Crawford** is Director of the College Centre for Quality Improvement, Royal College of Psychiatrists, London, UK.
